# Home based pulmonary tele-rehabilitation under telemedicine system for COPD: a cohort study

**DOI:** 10.1186/s12890-022-02077-w

**Published:** 2022-07-24

**Authors:** Ling Zhang, Ayiguli Maitinuer, Zhichuang Lian, Yafang Li, Wei Ding, Wenyi Wang, Chao Wu, Xiaohong Yang

**Affiliations:** 1 Graduate School, Xinjiang Medical University, Urumqi, 830001 China; 2 Department of Respiratory and Critical Care Medicine, People’s Hospital of Xinjiang Uygur Autonomous Region, No. 91 Tianchi Road, Tianshan District, Urumqi, 830001 China; 3 Xinjiang Clinical Research Center for Interstitial Lung Disease, Urumqi, 830001 China

**Keywords:** Telemedicine, Home based pulmonary rehabilitation, Chronic obstructive pulmonary disease, Effectiveness

## Abstract

**Background:**

Pulmonary tele-rehabilitation can improve adherence to pulmonary rehabilitation. However, there are few reports on home based pulmonary tele-rehabilitation. We assessed the effectiveness of home based pulmonary tele-rehabilitation under telemedicine system in patients with chronic obstructive pulmonary disease (COPD).

**Methods:**

This cohort study enrolled 174 patients with COPD who received home based pulmonary tele-rehabilitation under telemedicine system. The follow-up time was 12 weeks. Patients were grouped according to pulmonary rehabilitation weeks, number of rehabilitation times and total duration time, and when these three data were inconsistent, the two lowest values were grouped: control group (total rehabilitation weeks < 1 week, total number of rehabilitation times < 5, total duration time < 150 min, n = 46), pulmonary rehabilitation group 1 (PR-1) (1 week ≤ rehabilitation weeks < 4 weeks, 5 ≤ total number of rehabilitation times < 20, 150 min ≤ total duration time  < 1200 min, n = 31), pulmonary rehabilitation group 2 (PR-2) (4 weeks ≤ rehabilitation weeks < 8 weeks, 20 ≤ total number of rehabilitation times < 40, 600 min ≤ total duration time < 2400 min, n = 23), pulmonary rehabilitation group 3 (PR-3) (8 weeks ≤ rehabilitation weeks < 12 weeks, 40 ≤ total number of rehabilitation times < 60, 1200 min ≤ total duration time < 3600 min, n = 40) and pulmonary rehabilitation group 4 (PR-4) (rehabilitation weeks = 12 weeks, total number of rehabilitation times = 60, total duration time = 3600 min, n = 34). The clinical data before and after rehabilitation were collected and evaluated, including dyspnea symptoms, 6-min walk distance (6MWD), diaphragmatic mobility, anxiety and depression.

**Results:**

There was no significance difference between control group and PR-1 group. PR-2 group after rehabilitation had significantly decreased CAT and HAMA scores than control (P < 0.05). Compared with control, PR-3 group and PR-4 group after rehabilitation had significantly higher 6MWD and diaphragmatic motility during deep breathing, but significantly lower CAT score, mMRC score, HAMA score, and HAMD score (P < 0.05). Compared with before pulmonary rehabilitation, in PR-3 and PR-4 groups, the 6MWD and the diaphragmatic motility during deep breathing were significantly higher, while CAT score, mMRC score, HAMA score, and HAMD score (for PR-4 only) were significantly lower after pulmonary rehabilitation (P < 0.05). There was no significant difference between PR-3 group and PR-4 group (P > 0.05). In the 12-week pulmonary rehabilitation program, patients who completed at least 8 weeks, namely those in the PR-3 and PR-4 groups, accounted for 42.5% of the total number. Education, income and response rate to telemedicine system reminders were the main risk factors associated with home based pulmonary tele-rehabilitation.

**Conclusions:**

Home based pulmonary tele-rehabilitation under telemedicine system for more than 8 weeks can significantly improve the dyspnea symptoms, 6MWD, diaphragmatic mobility during deep breathing, and negative emotions of patients with moderate to severe stable COPD.

*Trial registration:* This study was registered at Chinese Clinical Trial Registry under registration number of ChiCTR2200056241CTR2200056241.

**Supplementary Information:**

The online version contains supplementary material available at 10.1186/s12890-022-02077-w.

## Background

Chronic obstructive pulmonary disease (COPD) is a chronic respiratory disease characterized by irreversible persistent airflow limitation. In the 2015 Global Burden of Disease Study, it is estimated that about 174.5 million adults worldwide suffer from COPD [1]. If spirometry-defined COPD is included, the disease burden may be as high as 384 million [2]. The overall prevalence of COPD in China is approximately 8.6% (95% CI 7.5–9.9) [3]. COPD is expected to become the leading cause of death globally in the next decade [3]. Pulmonary rehabilitation is recommended as one of the most cost-effective treatments for COPD [4]. However, in clinical practice, compliance for hospital-based pulmonary rehabilitation in CODP patients is poor, mainly due to inconvenience of transportation [5, 6]. A reliable home-based tele-rehabilitation may become a new standard for pulmonary rehabilitation in COPD [7].

Telemedicine platforms, mobile apps, or fitness bracelets can provide objective feedback on patient activity, but their effectiveness for home-based pulmonary tele-rehabilitation remains to be determined [8]. Several studies have reported that pulmonary tele-rehabilitation can improve self-control and reduce exacerbations, hospitalization rates, and length of stay in patients with COPD [9–11]. However, most studies were conducted in outpatient or community, and home based studies on pulmonary tele-rehabilitation are still limited [12, 13]. Moreover, there are still very few reports of pulmonary rehabilitation in China. Additionally, studies focusing on long-term home based tele-pulmonary rehabilitation (greater than 8 weeks) have small-scale. Thus, there is a pressing need for high-quality studies to enable the successful implementation of pulmonary rehabilitation at home and via telemedicine system.

Therefore, in this study, we conducted a cohort study to evaluate the effectiveness of home-based pulmonary tele-rehabilitation under telemedicine system in COPD. The telemedicine system was developed by China-Japan Friendship Hospital (Beijing) and Saike (Xiamen) Medical Equipment Co., Ltd. It has been tested for feasibility as well as functionality/user familiarity and has been successfully used in more than 30 tertiary hospitals including China-Japan Friendship Hospital. Our findings may provide certain reference for the use of telemedicine system in home-based pulmonary tele-rehabilitation for patients with COPD.

## Methods

### Trial design

This trial was designed as a cohort study and has been registered at Chinese Clinical Trial Registry under the registration number: ChiCTR2200056241.

### Ethics

This study was approved by the Ethics Committee of People’s Hospital of Xinjiang Uygur Autonomous Region and all methods were also performed in accordance with the relevant guidelines and regulations under the committee supervision. The signed informed consents were obtained from all participants.

### Participants

We enrolled patients with stable COPD in People’s Hospital of Xinjiang Uygur Autonomous Region from October 1, 2019 to June 1, 2021. Inclusion criteria: (1) COPD was diagnosed according to diagnostic criteria in 2019 GOLD (Global Initiative for Chronic Obstructive Lung Disease): Global Strategy for COPD Diagnosis, Treatment and Prevention [14]; (2) Patients with GOLD B-D; 3) Patients with normal cognition. Exclusion criteria: 1) Patients with serious physical and mental illness, including acute myocardial infarction within 6 months, unstable angina pectoris, malignant tumor, mental illness, etc.; (2) Patients with asthma, bronchiectasis, pulmonary fibrosis pulmonary tuberculosis, severe pulmonary bullae, pneumothorax and within 1 month after healing of pneumothorax, or history of previous lung surgery; (3) Patients with NRS2002 (Nutritional Risk Screening 2002) ≥ 3 points were excluded because these patients may have nutritional risk and need hospitalization for nutritional support [15]; 4) Patients with 6-min walk distance (6MWD) < 150 m.

### Home-based pulmonary tele-rehabilitation under telemedicine system

**Fig. 1:**
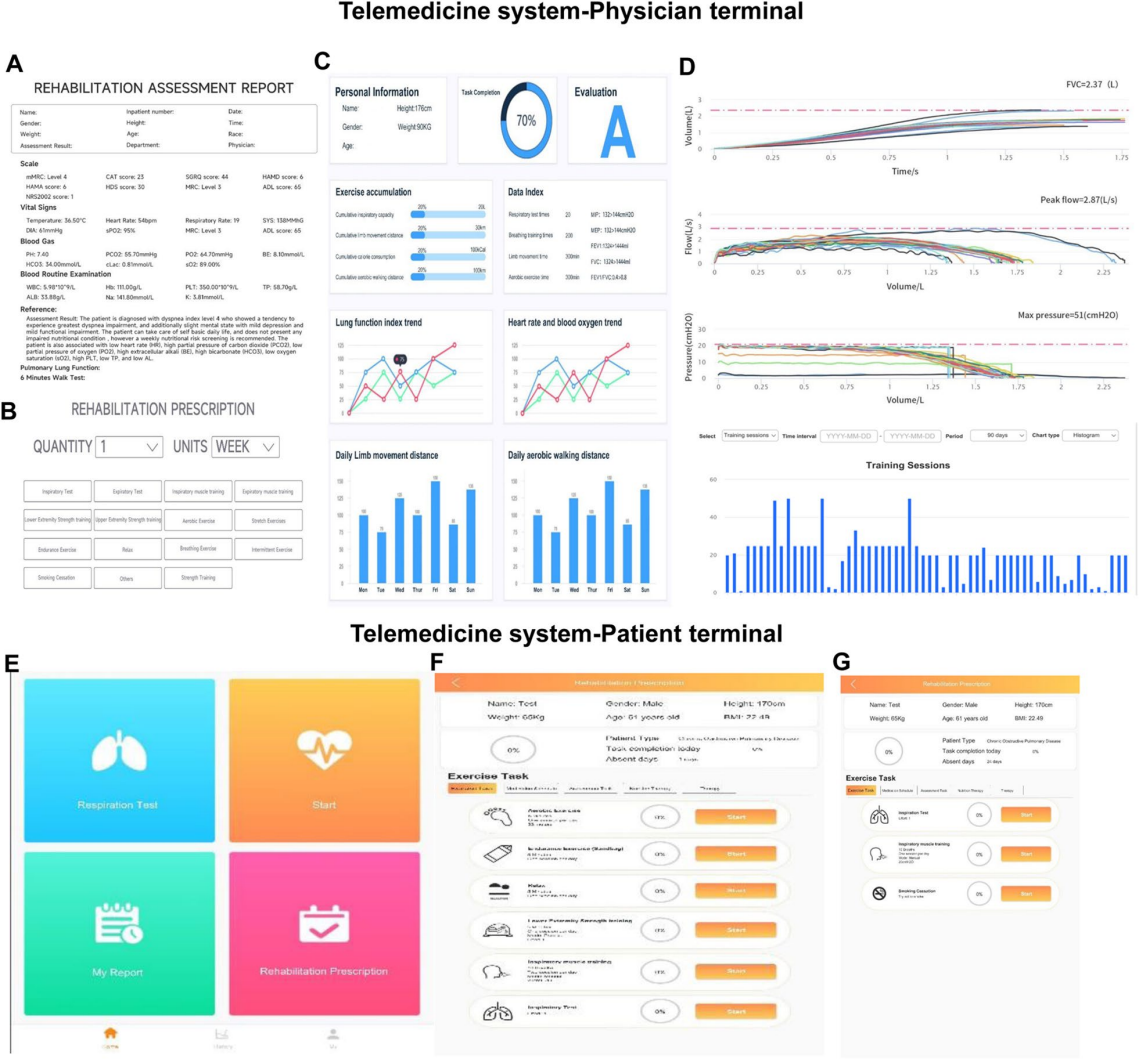
Telemedicine system. The telemedicine system consisted of a physician terminal and a patient terminal. **A** the pre-rehabilitation assessment report. **B** the rehabilitation prescription. **C** the rehabilitation training report. **D** the data of respiratory muscle training. **E** the main interface of the patient terminal. **F** the exercise prescription of the patient interface. **G** the patient terminal respiratory muscle training interface

All enrolled patients received training on pulmonary rehabilitation and received home based pulmonary tele-rehabilitation through the telemedicine system (https://m.xeek.cn/login/health.html). The telemedicine system consisted of a physician terminal and a patient terminal (Fig. 1). The physician terminal included the pre-rehabilitation assessment report (Fig. 1A), the rehabilitation prescription (Fig. 1B), the rehabilitation training report (Fig. 1C) and the data of respiratory muscle training (Fig. 1D). The pre-rehabilitation assessment report included basic information, 6MWD, and scores of CAT (COPD assessment test), mMRC (modified Medical Research Council), SGRQ (St. George’s Respiratory Questionnaire), HAMA (Hamilton anxiety rating scale), and HAMD (Hamilton depression rating scale). In the rehabilitation prescription, doctors could make exercise prescriptions and medication prescriptions, including exercise method and time, and medication type and time. In the rehabilitation training report, the doctor could view the patient's training times, exercise distance, heart rate during exercise, and lung function. The patient terminal included the patient terminal main interface (Fig. 1E), the patient terminal exercise prescription interface (Fig. 1F), and the patient terminal respiratory muscle training interface (Fig. 1G). The patients fed back on pulmonary rehabilitation through the mobile APP and completed the questionnaires regularly. Three attending physicians in our team checked the server once a day to monitor early warning messages, adverse events and completion of rehabilitation, etc. If early warning messages or adverse events are detected, early warning mechanism of the system will be triggered immediately, and early warning information will be sent to the doctor and the patient, and rehabilitation will be suspended at the same time. After receiving the warning information, the doctor will contact the patient by phone, inquire about the situation and determine whether it is necessary to conduct face-to-face consultation or whether the training should be continued. If the training should be continued, the prescription will be adjusted remotely on the physician terminal, and then the patient will be followed up by phone for 3 consecutive days. Dr. Yafang Li was responsible for contacting patients and conducting follow-up.

All pulmonary rehabilitation plans were made in the hospital by physicians or respiratory therapists. The physicians or respiratory therapists also trained the patients face to face on the use of the breathing device. The rehabilitation program was conducted under the guidance of the respiratory therapists or nurses for 2–3 times in the hospital. After that, the rehabilitation program was performed by the patients alone at home. Five times of pulmonary rehabilitation were conducted per week for 12 weeks. Each time lased for 1 h and contained 5 min of warm-up and relaxation, 10–15 min of respiratory muscle training with Breathing training device (XEEK, S2, China) (the optimal intensity was 30–50% cm H_2_O of maximum inspiratory or expiratory pressure), 25–30 min of aerobic exercise (brisk walking), and, 15–20 min of resistance exercise using elastic band or endurance training using sandbags (following videos with standard guidance on exercises). A suitable plan should meet the following standards: 1) target heart rate = (maximum heart rate—resting heart rate) × 60% + resting heart rate; 2) at least two of the following scores: Borg fatigue score 4–6, Brog shortness of breath score 12–14, and/or Brog6-20 scale score 13–16 [16]; 3) the oxygen saturation during exercise was not less than 90%. Pulmonary rehabilitation should be terminated if there is one of the following warning messages: (1) The vital signs were unstable, and the oxygen saturation was less than 90% in the resting state; (2) abnormal ECG monitoring, abnormal blood pressure (systolic blood pressure drop > 10 mmHg, ischemic symptoms, or excessive increase in blood pressure (≥ 250/115 mmHg)), and occurrence of new symptoms; (3) The patients requested to terminate the rehabilitation program.

### Clinical outcome

This study was conducted from October 1, 2019 to June 1, 2021, and the follow-up time was 12 weeks after inclusion. Data collection was performed by Dr. Ayiguli Maitinuer. Primary outcome was the improvement of 6MWD after 12 weeks. The secondary outcome was diaphragmatic mobility and dyspnea symptoms, which were assessed with mMRC [17]. The CAT was used to assess COPD health status, and the SGRQ was used to assess COPD health-related quality of life [18, 19]. Anxiety and depression of patients was evaluated with HAMA, and HAMD.

### Statistical analysis

SPSS 23.0 was used for statistical analysis. A two-sided P < 0.05 was considered statistically significant. Categorical data were expressed as frequencies and percentages and were compared by using chi-square test or Fisher's exact test. Measurement data of normal distribution were expressed as mean ± SD and compared with ANOVA (followed by LSD). Measurement data of non-normal distribution were presented as median (interquartile range) and compared by using Kruskal–Wallis rank sum test. Multivariate analysis was conducted by Logistic regression analysis.

## Results

### Baseline characteristics

**Fig. 2:**
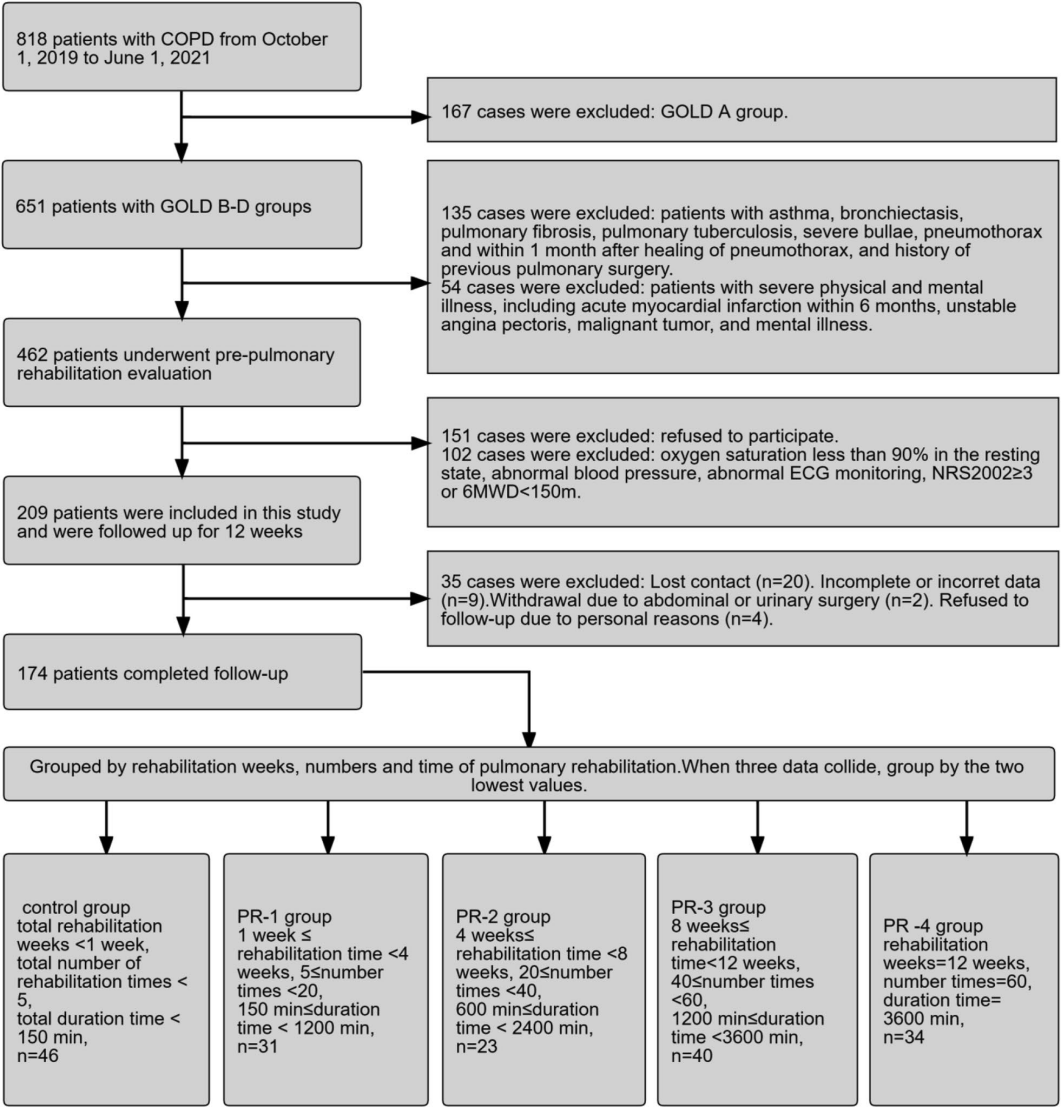
Study flowchart of participant enrollment. PR = pulmonary rehabilitation

A total of 209 patients with COPD were primarily enrolled, of which 35 patients were excluded due to loss of contact (n = 20), incomplete data (n = 9), or other reasons (n = 6; including 2 patients underwent surgery due to abdominal or urinary tract disease, and 4 patients refused follow-up due to personal reasons. As shown in Fig. 2, finally, 174 patients were included. They were divided into 5 groups according to pulmonary rehabilitation weeks, number of rehabilitation times and total duration time, and when these three data were inconsistent, the two lowest values were grouped: control group (total rehabilitation weeks < 1 week, total number of rehabilitation times < 5, total duration time < 150 min, n = 46), pulmonary rehabilitation group 1 (PR-1) (1 week ≤ rehabilitation weeks < 4 weeks, 5 ≤ number of rehabilitation times < 20, 150 min ≤ total duration time < 1200 min, n = 31), pulmonary rehabilitation group 2 (PR-2) (4 week ≤ rehabilitation weeks < 8 weeks, 20 ≤ number of rehabilitation times < 40, 600 min ≤ total duration time < 2400 min, n = 23), pulmonary rehabilitation group 3 (PR-3) (8 weeks ≤ rehabilitation weeks < 12 weeks, 40 ≤ number of rehabilitation times < 60, 1200 min ≤ total duration time < 3600 min, n = 40) and pulmonary rehabilitation group 4 (PR-4) (rehabilitation weeks = 12 weeks, total number of rehabilitation times = 60, total duration time = 3600 min, n = 34). No serious adverse events were reported during the study period. There was no significant difference in baseline characteristics among groups before rehabilitation, including age, sex, smoking history, disease severity, aCCI (age-adjusted Charlson comorbidity index), total number and total days of hospitalization for respiratory reasons in the past 1 year, 6MWD, CAT, mMRC, SGRQ, NRS2002, HAMA, HAMD, and, diaphragmatic mobility during rest and deep breathing (Table 1).

**Table 1:** Basic Data of patients with COPD before pulmonary rehabilitation

Variable	Control (n = 46)	PR-1 (n = 31)	PR-2 (n = 23)	PR-3 (n = 40)	PR-4 (n = 34)	*F /Z/*^*2*^	*P* values
Sex (Female, %)	25(54.3%)	16(51.6%)	13(56.5%)	18(45.0%)	15(44.1%)	1.64	0.801
Age (years)	63.7 ± 8.9	65.0 ± 7.4	63.1 ± 9.9	65.6 ± 8.1	62.6 ± 9.5	0.71	0.582
BMI(Kg/m^2^)	21.7 ± 5.9	27.5 ± 6.6	26.7 ± 5.8	27.6 ± 6.1	29.1 ± 5.8	0.68	0.604
Smoking history (yes)	7(15.2%)	4(12.9%)	4(17.4%)	7(17.5%)	3(8.8%)	1.54	0.838
FEV1, % predicted	42.7 ± 12.4	42.4 ± 11.9	46.1 ± 14.5	45.6 ± 12.5	43.3 ± 12.6	0.58	0.678
FEV1 severity						2.77	0.596
Moderate	12	6	9	12	9		
Severe and very severe	34	25	14	28	25		
aCCI(≥ 6)	16	14	8	16	11	1.48	0.829
Number of hospital respiratory admissions in past year (total)	46	29	15	37	29	3.20	0.525
Number of days of respiratory admissions past year (total)	346	226	112	277	206	3.58	0.465
6MWD (m)	538.0 ± 58.5	525.8 ± 50.9	548.2 ± 54.5	530.1 ± 56.1	533.3 ± 47.9	0.68	0.606
CAT	20.0 ± 4.4	20.2 ± 4.7	20.7 ± 4.6	20.4 ± 4.6	20.2 ± 4.0	0.09	0.983
mMRC	3(2,3)	3(2,3)	3(2,3)	3(2,3)	3(2,3)	3.89	0.420
SGRQ	23.2 ± 7.1	24.1 ± 5.1	21.7 ± 6.4	23.5 ± 6.4	23.7 ± 6.4	0.52	0.734
NRS2002	1(1,2)	1(1,2)	1(1,2)	1(1,2)	1(1,2)	0.40	0.982
HAMA	13(9,15.2)	12(9,15)	14(9,17)	13(9,17)	12(9,15)	2.50	0.645
HAMD	15(9.7,19)	15(9,19)	15(5,19)	12.5(5,17.7)	17(12.5,21.2)	5.93	0.205
Diaphragmatic mobility							
Diaphragmatic mobility during rest breathing (mm)	23.2 ± 2.8	22.9 ± 2.5	23.6 ± 2.3	22.7 ± 2.2	23.5 ± 2.8	0.68	0.603
Diaphragmatic mobility during deep breathing (mm)	43.7 ± 4.8	43.8 ± 4.3	45.5 ± 4.2	44.8 ± 5.1	45.0 ± 4.6	0.91	0.461

*PR *pulmonary rehabilitation, *FEV1 *forced expiratory volume in one second, *aCCI *age-adjusted Charlson comorbidity index, *6MWD *6-min walk distance, *CAT *COPD assessment test, *mMRC *modified Medical Research Council dyspnea score, *SGRQ *St. George’s Respiratory Questionnaire, *NRS2002 *nutritional risk screening 2002, *HAMA *Hamilton anxiety rating scale, *HAMD *Hamilton depression scale

### Effectiveness of home based pulmonary tele-rehabilitation

**Fig. 3:**
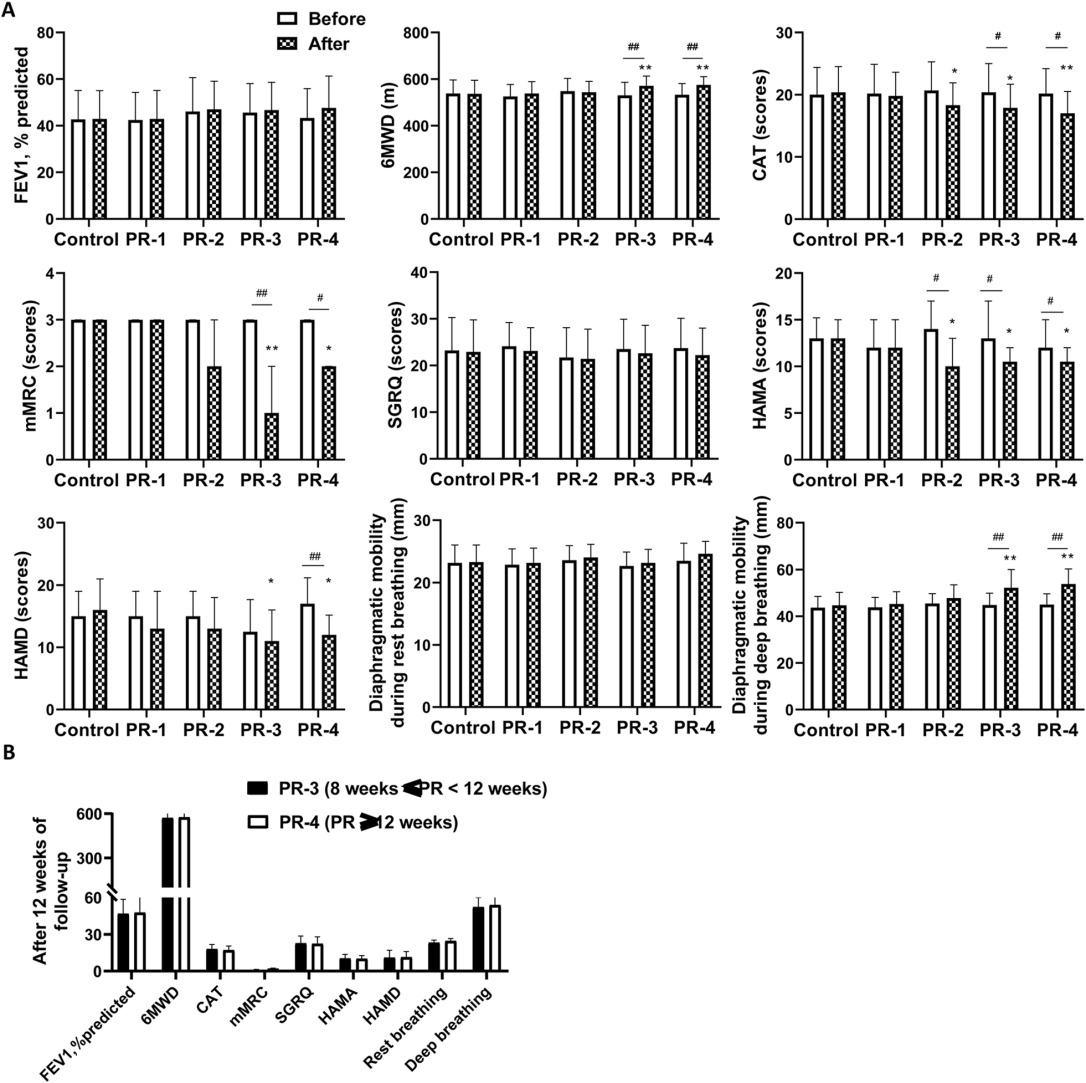
Changes of FEV1, 6MWD, CAT, mMRC, diaphragmatic mobility during deep/rest breathing, SGRQ, HAMA and HAMD in patients with COPD. **A** Changes of FEV1, 6MWD, CAT, mMRC, SGRQ, diaphragmatic mobility during deep/rest breathing, HAMA and HAMD in patients with COPD before and after pulmonary rehabilitation as well as between control group and PR-1/2/3/4 groups. *P < 0.05, **P < 0.01, compared to the control group. #P < 0.05, ##P < 0.01, compared with before pulmonary rehabilitation. **B** Changes of FEV1, 6MWD, CAT, mMRC, diaphragmatic mobility during deep/rest breathing, SGRQ, HAMA and HAMD between PR3 and PR4 groups. FEV1 = Forced expiratory volume in one second; 6MWD = 6-min walk distance; CAT = COPD assessment test; mMRC = modified Medical Research Council dyspnea score; SGRQ = St. George’s Respiratory Questionnaire; HAMA = Hamilton anxiety rating scale; HAMD = Hamilton depression scale

We then evaluated the effectiveness of home based pulmonary tele-rehabilitation. We first compared the data before and after pulmonary rehabilitation. As shown in Fig. 3A and Additional file 1: Table S1, in control, PR-1 and PR-2 groups, there was no significant difference in each index between before and after pulmonary rehabilitation. In PR-3 group, compared with before pulmonary rehabilitation, the 6MWD and the diaphragmatic motility during deep breathing were significantly higher, while CAT score, mMRC score, and HAMA score were significantly lower after pulmonary rehabilitation (P < 0.05). Similarly, in PR-4 group, compared with before pulmonary rehabilitation, the 6MWD and the diaphragmatic motility during deep breathing were significantly higher, while CAT score, mMRC score, HAMA score, and HAMD score were significantly lower after pulmonary rehabilitation (P < 0.05). No significant difference was found in SGRQ. In addition, we also compared the data after pulmonary rehabilitation between control group and PR-1/2/3/4 groups (Fig. 3A and Additional file 1: Table S1). The results showed that there were no differences in FEV1 (forced expiratory volume in the first second), 6MWD, CAT scores, mMRC scores, HAMA scores, HAMD scores, and diaphragmatic mobility between PR-1 group and control group (all P > 0.05). The CAT and HAMA scores in the PR-2 group after pulmonary rehabilitation were lower than that of control group (both P < 0.05). Compared with control group, the 6MWD and the diaphragmatic motility during deep breathing were significantly higher, while CAT score, mMRC score, HAMA score, and HAMD score were significantly lower in the PR-3 and PR-4 groups after pulmonary rehabilitation (all P < 0.05). Moreover, there were no significant differences in 6MWD, CAT score, mMRC score, HAMA score, HAMD score, and diaphragmatic mobility during deep breathing between PR-3 and PR-4 groups (Fig. 3B).

### Factors affecting home based pulmonary tele-rehabilitation in patients with COPD

In this study, the completion rate of 8-week home based pulmonary tele-rehabilitation under telemedicine system was 42.5% [(40 + 34)/174*100%], i.e. the percentage of subjects of the PR-3 and PR-4 groups in the total population. Then, we evaluated the factors affecting home based pulmonary tele-rehabilitation in patients with COPD. Place of residence, education, income, evaluation of telemedicine system and response rate to telemedicine system reminders were significantly associated with the completion of 8-week home based pulmonary tele-rehabilitation (all P < 0.05, Table 2). Multivariate analysis showed that education, income and response rate to telemedicine system reminders were significantly associated with efficacy of home based pulmonary tele-rehabilitation in patients with COPD (all P < 0.05, Table 3).

**Table 2:** Factors affecting home based pulmonary tele-rehabilitation in patients with COPD

Variable	PR < 8 weeks (n = 100)	PR ≥ 8 weeks (n = 74)	*Z/*^*2*^	*P* values
Lives alone (yes)	18(18.0%)	10(13.5%)	0.6	0.426
*Residence*			12.2	0.002**
City	40(40.0%)	49(66.2%)		
County	10(10.0%)	6(8.1%)		
Township	50(50%)	19(25.7%)		
*Current occupation*			1.2	0.269
On-the-job	32(32.0%)	18(24.3%)		
Retired or unemployed	68(68.0%)	56(75.7%)		
*Education (school years)*			6.1	0.047*
school years ≤ 9	44(44.0%)	31(41.9%)		
9 < school years ≤ 16	36(36.0%)	17(23.0%)		
school years > 16	20(20.0%)	26(35.1%)		
*Income(per month)*			10.7	0.005*
≥ 1008$	7(7.0%)	14(18.9%)		
388–1800$	70(70.0%)	54(73.0%)		
< 388$	23(23.0%)	6(8.1%)		
*Self-care ability*			4.0	0.258
Completely independent	15(15.0%)	9(12.2%)		
Partly dependent	39(39.0%)	40(54.1%)		
Mostly dependent	40(40.0%)	21(28.4%)		
Totally dependent	6(6.0%)	4(5.4%)		
*Evaluation of telemedicine system*			6.9	0.031*
Useful	32(32.0%)	38(51.4%)		
Not sure	26(26.0%)	16(21.6%)		
Useless	42(42.0%)	20(27.0%)		
*Response rate to telemedicine system reminders*			42.5	0.001**
High(≥ 66%)	29(29.0%)	58(78.4%)		
Moderate(33–66%)	52(52.0%)	9(12.2%)		
Low(< 33%)	19(19.0%)	7(9.5%)		

*PR *pulmonary rehabilitation

*P < 0.05,**P < 0.01, compared with the group of PR < 8 weeks. Response rate to telemedicine system reminders means the patient’s response to the reminder information issued by the telemedicine system, if the patient’s response rate accounts for 66% of the total number of reminders, it is considered to be high, 33–66% is general, less than 33% is low response rate

**Table 3:** Logistic regression analysis of factors affecting home based pulmonary tele-rehabilitation in patients with COPD

Variable	B values	Standard error	Wald ^*2*^ values	*OR* values	95% CI	*P* values
Place of residence	0.10	0.24	0.17	1.11	0.69–1.76	0.676
Education (school years)	0.53	0.25	4.52	1.71	1.04–2.79	0.033*
Income (per month)	1.13	0.37	9.55	3.09	1.51–6.32	0.002**
Evaluation of telemedicine system	0.37	0.21	3.10	1.45	0.96–2.20	0.078
Response rate to telemedicine system reminders	1.65	0.33	24.43	5.21	2.71–10.02	0.001**

^*^P < 0.05,**P < 0.01

## Discussion

Pulmonary rehabilitation has been recommended as an effective treatment for COPD. However, to date, most of the evidence of pulmonary rehabilitation is from hospital-based studies, while the evidence for home-based tele-rehabilitation is still limited [7, 20]. Furthermore, for studies focusing on long-term home pulmonary tele-rehabilitation (greater than 8 weeks) [21–24], the sample size was relatively small and various tele-rehabilitation methods were used [21–24]. Thus, development of novel pulmonary rehabilitation management methods, including home-based models and the use of telemedicine system, can help increase patient compliance and improve the efficiency of pulmonary rehabilitation. In this study, we explored the effectiveness of home based pulmonary rehabilitation using a telemedicine system. The telemedicine system was composed of a physician terminal and a patient terminal. It had the functions of automatic notification, physician–patient communication, dynamic display of follow-up data, and automatic warning of abnormal indicators, and could track and manage patients in real time for home based pulmonary tele-rehabilitation.

We found that after home based pulmonary tele-rehabilitation under telemedicine system for 8–12 weeks, patients with COPD had significant improvement in 6MWD, dyspnea symptoms, diaphragmatic mobility during deep breathing, anxiety and depression, suggesting that home-based pulmonary rehabilitation results in significantly greater improvements in exercise capacity and health-related quality of life in patients with COPD. Our results was consistence with previous studies, which showed that home based pulmonary rehabilitation via phone or videoconferencing could significantly improve exercise tolerance, dyspnea, and quality of life in patients with COPD [25, 26]. However, the HAMD score was significantly improved until 12 weeks, suggesting that the effect of pulmonary rehabilitation in improving depression may be more obvious until to 12 weeks. The results of this study were also consistent with the other previous findings [27, 28], which showed that pulmonary rehabilitation could effectively reduce anxiety and depression, and had better effect on depression if there is longer duration. In addition, we found that pulmonary rehabilitation only improved diaphragmatic mobility during deep breathing, but had no effect on diaphragmatic mobility during rest breathing. During deep breathing, the need for diaphragm strength is greatly increased [29], and the effect of pulmonary rehabilitation on diaphragmatic mobility during this period may be related to improved diaphragm muscle strength and exercise capacity. Decreased diaphragmatic mobility in patients with COPD is associated with lung hyperinflation, air trapping, decreased strength, and muscle fiber loss [30]. During rest breathing, the diaphragm provides 75% of the lung volume. In patients with COPD, the motility of the diaphragm is significantly reduced due to lung hyperinflation [31]. The observation that pulmonary rehabilitation did not improve the motility of the diaphragm during rest breathing may be because pulmonary rehabilitation could not improve lung hyperinflation [32].

In this study, the completion rate of 8-week home based pulmonary tele-rehabilitation under telemedicine system was 42.5%, and income, education and the lower response rate of the telemedicine system were found to be the main factors affecting telemedicine in the pulmonary rehabilitation. Low income of patients with COPD may affect their willingness or ability to participate in pulmonary tele-rehabilitation, but in the long run, pulmonary tele-rehabilitation may actually reduce their medical costs. Recently, a meta-analysis showed that pulmonary rehabilitation could reduce the risk of admission to the hospital due to respiratory disease [33]. Treatment cost of COPD due to aggravation accounted for 70% of the total therapeutic cost of the disease [34, 35]. In China, the direct medical cost of COPD are $ 72 to $ 3565 per year, the indirect cost is $ 20 to $ 783 a year, and the total cost is approximately $ 700–1800 [36]. In theory, the main cost of the telemedicine management system is the cost of establishing a system in the initial period. However, the system is designed for long-term use, and thus the benefits of pulmonary rehabilitation in the medium and long-term use of telemedicine management system may be sufficient to produce favorable cost-effectiveness. In addition, low education levels may affect patients' willingness to accept a new model of pulmonary rehabilitation and the ability to operate APPs, which may lead to lower response rates in telemedicine systems. During the follow-up period, by tracking the patients' pulmonary rehabilitation status and early warning messages in real time, there were no adverse events caused by pulmonary rehabilitation, suggesting that home based pulmonary tele-rehabilitation under the management of the telemedicine system is safe.

There are some limitations in this study. First, a control group that did not use telemedicine was not set up due to insufficient patient number. Second, critically ill and poorly nourished patients with COPD were not included. Therefore, the results of this study cannot be applied to these patients. Third, the duration of follow-up time was 12 weeks, which was not enough for evaluation of long-term prognosis, and longer follow-up is required. Fourth, the web/technology literacy of the participants were not analyzed in this study. Fifth, one-on-one video supervision of patients was not performed. Thus, the accuracy of the exercises cannot be guaranteed. Further studies are warranted.

## Conclusions

To sum up, home based pulmonary tele-rehabilitation under telemedicine system for more than 8 weeks can significantly improve the 6MWD, symptoms, diaphragmatic mobility, anxiety and depression of patients with moderate to severe stable COPD. Home based pulmonary tele-rehabilitation under telemedicine system may be used as an effective treatment in patients with COPD.

## Supplementary Information

**Additional file 1: Table S1.** Changes of FEV1, 6MWD, CAT,mMRC,diaphragmatic mobility during deep/rest breathing, SGRQ, HAMAand HAMD in patients with COPD.

## Data Availability

The datasets generated and/or analysed during the current study are not publicly available due to local ownership of the data but are available from the corresponding author on reasonable request.

## Abbreviations

COPD: Chronic obstructive pulmonary disease
CAT: COPD assessment test
mMRC: Modified Medical Research Council
SGRQ: St. George’s Respiratory Questionnaire
HAMA: Hamilton anxiety rating scale
HAMD: Hamilton depression scale
6MWD: 6-Minute walk distance
PR-1: Pulmonary rehabilitation group 1

## References

[1] Vogelmeier CF, Criner GJ, Martinez FJ, Anzueto A, Barnes PJ, Bourbeau J, Celli BR, Chen R, Decramer M, Fabbri LM (2017). Global strategy for the diagnosis, management, and prevention of chronic obstructive lung disease 2017 report. Gold executive summary. Am J Respir Crit Care Med.

[2] Adeloye D, Chua S, Lee C, Basquill C, Papana A, Theodoratou E, Nair H, Gasevic D, Sridhar D, Campbell H (2015). Global and regional estimates of COPD prevalence: systematic review and meta-analysis. J Glob Health.

[3] Wang C, Xu J, Yang L, Xu Y, Zhang X, Bai C, Kang J, Ran P, Shen H, Wen F (2018). Prevalence and risk factors of chronic obstructive pulmonary disease in China (the China Pulmonary Health [CPH] study): a national cross-sectional study. Lancet.

[4] Fiorentino G, Esquinas AM, Annunziata A (2020). Exercise and chronic obstructive pulmonary disease (COPD). Adv Exp Med Biol.

[5] Mathar H, Fastholm P, Lange P, Larsen NS (2017). Why do patients decline participation in offered pulmonary rehabilitation? A qualitative study. Clin Rehabil.

[6] Sahin H, Naz I (2018). Why are COPD patients unable to complete the outpatient pulmonary rehabilitation program?. Chron Respir Dis.

[7] Tsutsui M, Gerayeli F, Sin DD (2021). Pulmonary rehabilitation in a Post-COVID-19 world: telerehabilitation as a new standard in patients with COPD. Int J Chron Obstruct Pulmon Dis.

[8] Barbosa MT, Sousa CS, Morais-Almeida M, Simoes MJ, Mendes P (2020). Telemedicine in COPD: an overview by topics. COPD.

[9] Hansen H, Bieler T, Beyer N, Kallemose T, Wilcke JT, Ostergaard LM, Frost Andeassen H, Martinez G, Lavesen M, Frolich A (2020). Supervised pulmonary tele-rehabilitation versus pulmonary rehabilitation in severe COPD: a randomised multicentre trial. Thorax.

[10] Lilholt PH, Witt Udsen F, Ehlers L, Hejlesen OK (2017). Telehealthcare for patients suffering from chronic obstructive pulmonary disease: effects on health-related quality of life: results from the Danish 'TeleCare North' cluster-randomised trial. BMJ Open.

[11] Bhatt SP, Patel SB, Anderson EM, Baugh D, Givens T, Schumann C, Sanders JG, Windham ST, Cutter GR, Dransfield MT (2019). Video telehealth pulmonary rehabilitation intervention in chronic obstructive pulmonary disease reduces 30-Day readmissions. Am J Respir Crit Care Med.

[12] Vasilopoulou M, Papaioannou AI, Kaltsakas G, Louvaris Z, Chynkiamis N, Spetsioti S, Kortianou E, Genimata SA, Palamidas A, Kostikas K (2017). Home-based maintenance tele-rehabilitation reduces the risk for acute exacerbations of COPD, hospitalisations and emergency department visits. Euro Respir J.

[13] Bernocchi P, Vitacca M, La Rovere MT, Volterrani M, Galli T, Baratti D, Paneroni M, Campolongo G, Sposato B, Scalvini S (2018). Home-based telerehabilitation in older patients with chronic obstructive pulmonary disease and heart failure: a randomised controlled trial. Age Ageing.

[14] Singh D, Agusti A, Anzueto A, Barnes PJ, Bourbeau J, Celli BR, Criner GJ, Frith P, Halpin DMG, Han M (2019). Global strategy for the diagnosis, management, and prevention of chronic obstructive lung disease: the GOLD science committee report 2019. Euro Respir J.

[15] Kondrup J, Allison SP, Elia M, Vellas B, Plauth M (2003). Educational, clinical practice committee ESoP, Enteral N: **ESPEN **guidelines for nutrition screening 2002. Clin Nutr.

[16] Gloeckl R, Schneeberger T, Jarosch I, Kenn K (2018). Pulmonary rehabilitation and exercise training in chronic obstructive pulmonary disease. Deutsch Arzteblatt Int.

[17] Cheng SL, Lin CH, Wang CC, Chan MC, Hsu JY, Hang LW, Perng DW, Chong-Jen Y, Wang HC (2019). Comparison between COPD assessment test (CAT) and modified medical research council (mMRC) dyspnea scores for evaluation of clinical symptoms, comorbidities and medical resources utilization in COPD patients. J Formos Med Assoc.

[18] Sciriha A, Lungaro-Mifsud S, Scerri J, Magro R, Camilleri L, Montefort S (2017). Health status of COPD patients undergoing pulmonary rehabilitation: a comparative responsiveness of the CAT and SGRQ. Chron Respir Dis.

[19] Choi JY, Yoon HK, Shin KC, Park SY, Lee CY, Ra SW, Jung KS, Yoo KH, Lee CH, Rhee CK (2019). CAT score and SGRQ definitions of chronic bronchitis as an alternative to the classical definition. Int J Chron Obstruct Pulmon Dis.

[20] Cox NS, Dal Corso S, Hansen H, McDonald CF, Hill CJ, Zanaboni P, Alison JA, O'Halloran P, Macdonald H, Holland AE (2021). Telerehabilitation for chronic respiratory disease. Cochrane Database Syst Rev.

[21] Holland AE, Mahal A, Hill CJ, Lee AL, Burge AT, Cox NS, Moore R, Nicolson C, O'Halloran P, Lahham A (2017). Home-based rehabilitation for COPD using minimal resources: a randomised, controlled equivalence trial. Thorax.

[22] Horton EJ, Mitchell KE, Johnson-Warrington V, Apps LD, Sewell L, Morgan M, Taylor RS, Singh SJ (2018). Comparison of a structured home-based rehabilitation programme with conventional supervised pulmonary rehabilitation: a randomised non-inferiority trial. Thorax.

[23] Tousignant M, Marquis N, Page C, Imukuze N, Metivier A, St-Onge V, Tremblay A (2012). In-home telerehabilitation for older persons with chronic obstructive pulmonary disease: a pilot study. Int J Telerehabil.

[24] Marquis N, Larivee P, Saey D, Dubois MF, Tousignant M (2015). In-Home pulmonary telerehabilitation for patients with chronic obstructive pulmonary disease: a pre-experimental study on effectiveness, satisfaction, and adherence. Telemed J e-health Off J Am Telemed Assoc.

[25] Paneroni M, Colombo F, Papalia A, Colitta A, Borghi G, Saleri M, Cabiaglia A, Azzalini E, Vitacca M (2015). Is Telerehabilitation a safe and viable option for patients with COPD? A feasibility study. COPD.

[26] Tsai LL, McNamara RJ, Moddel C, Alison JA, McKenzie DK, McKeough ZJ (2017). Home-based telerehabilitation via real-time videoconferencing improves endurance exercise capacity in patients with COPD: the randomized controlled TeleR Study. Respirology.

[27] Gordon CS, Waller JW, Cook RM, Cavalera SL, Lim WT, Osadnik CR (2019). Effect of pulmonary rehabilitation on symptoms of anxiety and depression in COPD: a systematic review and meta-analysis. Chest.

[28] Ozturk BO, Alpaydin AO, Ozalevli S, Guler N, Cimilli C (2020). Self-management training in chronic obstructive lung disease improves the quality of life. Turk Thorac J.

[29] Corbellini C, Boussuges A, Villafañe JH, Zocchi L (2018). Diaphragmatic mobility loss in subjects with moderate to very severe COPD may improve after in-patient pulmonary rehabilitation. Respir Care.

[30] Paulin E, Yamaguti WP, Chammas MC, Shibao S, Stelmach R, Cukier A, Carvalho CR (2007). Influence of diaphragmatic mobility on exercise tolerance and dyspnea in patients with COPD. Respir Med.

[31] Klimathianaki M, Vaporidi K, Georgopoulos D (2011). Respiratory muscle dysfunction in COPD: from muscles to cell. Curr Drug Targets.

[32] Polkey MI, Qiu ZH, Zhou L, Zhu MD, Wu YX, Chen YY, Ye SP, He YS, Jiang M, He BT (2018). Tai chi and pulmonary rehabilitation compared for treatment-naive patients with COPD: a randomized controlled trial. Chest.

[33] Jenkins AR, Gowler H, Curtis F, Holden NS, Bridle C, Jones AW (2018). Efficacy of supervised maintenance exercise following pulmonary rehabilitation on health care use: a systematic review and meta-analysis. Int J Chron Obstruct Pulmon Dis.

[34] Byng D, Lutter JI, Wacker ME, Jorres RA, Liu X, Karrasch S, Schulz H, Vogelmeier C, Holle R (2019). Determinants of healthcare utilization and costs in COPD patients: first longitudinal results from the German COPD cohort COSYCONET. Int J Chron Obstruct Pulmon Dis.

[35] Iheanacho I, Zhang S, King D, Rizzo M, Ismaila AS (2020). Economic burden of chronic obstructive pulmonary disease (COPD): a systematic literature review. Int J Chron Obstruct Pulmon Dis.

[36] Zhu B, Wang Y, Ming J, Chen W, Zhang L (2018). Disease burden of COPD in China: a systematic review. Int J Chron Obstruct Pulmon Dis.

