# Supplementary material for: Home based pulmonary tele-rehabilitation under telemedicine system for COPD: a cohort study
Source: BMC Pulm Med. 2022 Jul 24;22:284. doi: 10.1186/s12890-022-02077-w (PMC9310454; doi:10.1186/s12890-022-02077-w)
Supplement: Supplementary file 1 — Additional file 1: Table S1. Changes of FEV1, 6MWD, CAT,mMRC,diaphragmatic mobility during deep/rest breathing, SGRQ, HAMAand HAMD in patients with COPD. [file 12890_2022_2077_MOESM1_ESM.docx]

**Supplementary Table 1. Changes of FEV1, 6MWD, CAT, mMRC, diaphragmatic mobility during deep/rest breathing, SGRQ, HAMA and HAMD in patients with COPD.**

| Variable | Control  (n=46)l | PR-1  (n=31) | PR-2  (n=23) | PR-3  (n=40) | PR-4  (n=34) | *Z/F* | *P* values |
| --- | --- | --- | --- | --- | --- | --- | --- |
| FEV1, % predicted | 42.9±12.1 | 42.9±12.2 | 47.0±12.1 | 46.7±11.9 | 47.6±13.7 | 1.25 | 0.289 |
| 6MWD (m) | 536.9±58.2 | 538.0±51.3 | 543.9±46.7 | 570.7±42.1^ab^ | 575.3±35.2^ab^ | 5.42 | <0.001** |
| CAT | 20.4±4.1 | 19.8±3.8 | 18.3±3.6^a^ | 17.9±3.8^ab^ | 17.0±3.5^ab^ | 4.94 | <0.001** |
| mMRC | 3(2,3) | 3(2,3) | 2(2,3) | 1(1,2) ^ab^ | 2(1,2) ^ab^ | 59.44 | <0.001** |
| SGRQ | 22.9±6.9 | 23.1±5.0 | 21.4±6.4 | 22.6±6.0 | 22.2±5.8 | 0.33 | 0.856 |
| HAMA | 13(9,15) | 12(9,15) | 10(7,13) ^ab^ | 10.5(7,12) ^ab^ | 10.5(9,12) ^ab^ | 12.22 | 0.016* |
| HAMD | 16(9,21) | 13(9,19) | 13(5,18) | 11(5,16)^b^ | 12(8.7,15.2)^ab^ | 9.93 | 0.042* |
| Diaphragmatic mobility |  |  |  |  |  |  |  |
| Diaphragmatic mobility during rest breathing (mm) | 23.3±2.7 | 23.2±2.3 | 24.0±2.1 | 23.2±2.1 | 24.6±2.0 | 2.27 | 0.063 |
| Diaphragmatic mobility during deep breathing (mm) | 44.7±5.6 | 45.3±5.3 | 47.8±5.7 | 52.2±7.8^ab^ | 53.8±6.5^ab^ | 15.39 | <0.001** |

Note：**P*＜0.05,***P*＜0.01, compared between 5 groups. a represents P<0.05 compared with the control group, and b represents P<0.05 compared with before pulmonary rehabilitation.

FEV1= forced expiratory volume in one second; aCCI=age-adjusted Charlson Comorbidity Index; 6MWD = 6-min walk distance; CAT=COPD assessment test; mMRC = modified Medical Research Council dyspnea score; SGRQ = St. George’s Respiratory Questionnaire; NRS2002=Nutritional risk screening 2002; HAMA=Hamilton anxiety rating scale; HAMD=Hamilton depression scale.
